# Novel Multimodal Imaging System for High-Resolution and High-Contrast Tissue Segmentation Based on Chemical Properties

**DOI:** 10.3390/s25206342

**Published:** 2025-10-14

**Authors:** Björn van Marwick, Felix Lauer, Felix Wühler, Miriam Rittel, Carmen Wängler, Björn Wängler, Carsten Hopf, Matthias Rädle

**Affiliations:** 1 CeMOS Research and Transfer Center, Mass Spectrometry and Optical Spectroscopy, Technische Hochschule Mannheim, 68163 Mannheim, Germany; 2 Medical Faculty Mannheim, Heidelberg University, 68167 Mannheim, Germany; 3 Biomedical Chemistry, Clinic of Radiology and Nuclear Medicine, Medical Faculty Mannheim, Heidelberg University, 68167 Mannheim, Germany; 4 Research Campus M^2^OLIE, Medical Faculty Mannheim, Heidelberg University, 68167 Mannheim, Germany; 5 Molecular Imaging and Radiochemistry, Clinic of Radiology and Nuclear Medicine, Medical Faculty Mannheim, Heidelberg University, 68167 Mannheim, Germany; 6 Mannheim Institute for Intelligent Systems in Medicine (MIISM), Medical Faculty Mannheim, Heidelberg University, 68167 Mannheim, Germany

**Keywords:** multimodal imaging, mid-infrared microscopy, fluorescence microscopy, label-free diagnostics, tissue characterization, chemical specificity, mouse brain imaging, spectral clustering, biomedical optics

## Abstract

**Highlights:**

**What are the main findings?**

- Development of a multimodal imaging system combining mid-infrared (MIR) and high-resolution autofluorescence microscopy for label-free tissue analysis.
- Enables reliable segmentation of brain and skin tissue regions, resolving substructures in the 5–20 µm range and differentiating lipid-rich white matter from protein-rich gray matter.
- Demonstrates that multimodal clustering achieves comparable accuracy to high-resolution FTIR imaging while drastically reducing the acquisition time.

**What is the implication of the main finding?**

- Provides enhanced spatial and chemical information without external labeling, improving the relationship between speed, resolution, and analytical accuracy.
- Offers a time-efficient approach for detailed tissue characterization, with applications in biomedical research and rapid, label-free diagnostics.

**Abstract:**

Accurate and detailed tissue characterization is a central goal in medical diagnostics, often requiring the combination of multiple imaging modalities. This study presents a multimodal imaging system that integrates mid-infrared (MIR) scanning with fluorescence imaging to enhance the chemical specificity and spatial resolution in biological samples. A motorized mirror allows rapid switching between MIR and fluorescence modes, enabling efficient, co-registered data acquisition. The MIR modality captures label-free chemical maps based on molecular vibrations, while the fluorescence channel records endogenous autofluorescence for additional biochemical contrast. Applied to mouse brain tissue, the system enabled the clear differentiation of gray matter and white matter, supported by the clustering analysis of spectral features. The addition of autofluorescence imaging further improved anatomical segmentation and revealed fine structural details. In mouse skin, the approach allowed the precise mapping of the layered tissue architecture. These results demonstrate that combining MIR scanning and fluorescence imaging provides complementary, label-free insights into tissue morphology and chemistry. The findings support the utility of this approach as a powerful tool for biomedical research and diagnostic applications, offering a more comprehensive understanding of tissue composition without relying on staining or external markers.

## 1. Introduction

The precise, non-invasive characterization of biological tissue is essential for many medical applications, including diagnostics, pathology, intraoperative decision making, and pharmaceutical development. Differentiating between tissue types, such as healthy, inflamed, or neoplastic structures, is critical for early detection and accurate treatment planning. Traditional histological analysis, although highly specific, is invasive, time-consuming, and unsuitable for real-time use. As a result, there is an increasing demand for imaging techniques that provide both morphological and chemical information without the need for labeling or physical sectioning and in very short acquisition times. In this context, the combination of complementary, label-free optical methods offers a promising path forward [1,2,3].

In medical diagnostics, the integration of multiple methods is often crucial in ensuring diagnostic accuracy. Combining different techniques into a single device results in a multimodal measurement system, which offers various advantages, including a higher spatial resolution, molecular-level biological information with increased sensitivity, and the ability to visualize sample details that conventional imaging methods cannot adequately capture. Techniques are often combined in such a way that the limitations of individual methods do not significantly affect the overall system’s performance. Examples of such combinations include Raman and fluorescence measurements with visual or near-infrared imaging, as well as integration with computed tomography (CT) or ultrasound techniques [4,5,6,7].

### 1.1. Conventional and Established Imaging Methods

Most contemporary imaging techniques used for biological sample analysis are non-invasive, meaning that they do not alter the chemical or structural integrity of the specimen. However, certain widely applied methods, such as conventional light microscopy and fluorescence microscopy, often rely on the application of contrast agents or molecular markers. These exogenous substances can modify the chemical composition and physical properties of the tissue, introducing fluorescence or enhancing the contrast to enable more detailed visualization. A major drawback of this approach is the potential loss of sample integrity for subsequent analyses. A prominent example of an invasive technique is hematoxylin and eosin (H&E) staining, which enables the histological differentiation of nuclei and cytoplasmic structures [8,9,10].

Alternatively, fluorescence-based methods employ labeled ligands, such as antibody conjugates or nucleic acid probes (e.g., diamidino-phenyl-indole (DAPI) for deoxyribonucleic acid (DNA), phalloidin-FITC for actin) to selectively bind and visualize specific biomolecules. While fluorescent dyes enhance the signal strength and enable high molecular specificity, this approach is inherently limited to predefined targets and relies on sufficient labeling efficiency. In some cases, intrinsic tissue autofluorescence can overlap with dye emission spectra and thereby reduce the contrast and image quality. Nevertheless, fluorescence imaging remains an important technique, particularly when high specificity and molecular information are required. In this context, fluorescence-based methods and approaches for determining the overall chemical composition should be considered as complementary tools, with their relative strengths depending on the specific application or research question. Despite its advantages of a high lateral resolution (~200 nm) and rapid acquisition times, this method is invasive [11,12,13,14].

Fourier transform infrared spectroscopy (FTIR) is a non-invasive technique that provides chemical contrast based on molecular vibrational bands. By recording full MIR spectra at each pixel and analyzing both the peak position and intensity, it is possible to map chemical distributions across the sample. However, FTIR is limited by long acquisition times (~30 min/cm^2^), a relatively low spatial resolution (~6.25 µm), and the need for reflective or IR-enhanced substrates to achieve sufficient signal contrast [9,15,16,17].

Raman microspectroscopy offers complementary chemical information by detecting inelastically scattered light resulting from vibrational transitions. Compared to FTIR, Raman bands are often more specific and less affected by background signals from complex surfaces. However, Raman imaging suffers from very slow acquisition speeds, low sensitivity, and a limited resolution. More critically, the high excitation powers required to generate measurable Raman signals can induce significant local heating. This may lead to thermal damage or even visible burning of the sample, rendering the method invasive under many conditions [18,19].

Matrix-assisted laser desorption/ionization (MALDI) enables the highly specific detection of molecular structures at the compound class level, offering more detailed information than conventional vibrational spectroscopy. However, it requires complex sample preparation and analysis, causes irreversible sample destruction, and is limited by long acquisition times, high costs, and restricted spatial coverage, making it suitable mainly for targeted analysis after preliminary imaging [20,21,22].

Multimodal imaging systems represent a promising strategy to overcome some of these limitations by combining complementary imaging modalities into a single integrated setup. A notable example is the system developed by Heintz et al. (2021), designed for distinguishing gray and white matter in brain tissue [18]. This system integrates brightfield, darkfield, and polarization microscopy with visual and near-infrared (VIS/NIR) reflectance and Raman spectroscopy, enabling simultaneous morphological and chemical characterization. Rapid modalities can identify regions of interest (ROIs), which can then be analyzed in detail using slower, more specific methods such as Raman spectroscopy. While this approach offers considerable advantages in terms of analytical flexibility and data richness, it is not without limitations. The Raman component, in particular, remains a significant bottleneck due to long measurement times and limited spatial resolutions. Moreover, the high laser power required for excitation can induce local thermal damage, especially in sensitive biological tissues, compromising sample integrity. Thus, although most modalities in such systems are non-destructive, the Raman subsystem must be regarded as invasive under practical conditions [18,23].

### 1.2. Multimodal Imaging Approach for Tissue Analysis

To overcome individual limitations and combine short acquisition times, high contrast, high spatial resolutions, and complementary chemical information, this study pursues a multimodal imaging approach within a single integrated system. To meet the requirement of non-invasiveness, fluorescence imaging based on intrinsic autofluorescence is combined with a novel, label-free mid-infrared (MIR) scanning technique [9,17,24,25].

Mid-infrared (MIR) spectroscopy is based on the interaction of infrared radiation with molecular vibrations in a sample. When infrared light passes through or reflects off a biological tissue, specific wavelengths are absorbed depending on the vibrational modes of chemical bonds present in the molecules. Each functional group, such as C–H, N–H, C=O, or O–H, has characteristic absorption bands in the MIR region (typically 4000 to 400 cm^−1^), allowing for molecular identification and structural analysis. The fundamental principle governing this interaction is Beer–Lambert’s law, which relates absorbance *A* to the concentration *c* of absorbing species, the path length *l*, and the molar absorptivity *ε* [26,27,28].

*A* = *ε*
*⋅c*
*⋅l* (1)

In biological tissues, the absorbance spectrum represents a complex mixture of overlapping signals from proteins, lipids, nucleic acids, and water [24,29]. By selecting specific wavenumbers and using narrowband sources, it becomes possible to emphasize particular molecular features (e.g., the amide I band with proteins or CH-stretching bands with lipids) [16,24].

This study uses a scanning system with discrete MIR wavelengths to map chemical components in tissue sections at a micrometer resolution. By measuring absorbance point by point, it enables both structural imaging and biochemical differentiation, such as distinguishing gray from white matter. The confocally designed MIR scanning system employs precisely selected mid-infrared wavenumbers (2–4 μm) to visualize lipid- and protein-containing substances with a high spatial resolution (20 μm) and rapid acquisition speed (3 s/cm^2^/laser). By focusing on diagnostically relevant absorption bands, the system provides analytically comparable results for the evaluation of tissue sections, despite its narrower spectral range compared to FTIR. The described scanning system was developed in previous studies, where it was shown to support both pathological assessment and the investigation of tissue samples in contexts such as drug development. In addition, it has been applied to define regions of interest (ROIs) as a preparatory step for MALDI analyses, thereby facilitating a targeted workflow for molecular characterization. In these applications, the system successfully distinguished gray from white matter in tissue sections, underscoring its ability to resolve structural and biochemical differences in complex biological samples [9,16,17,24,25].

Such differentiation is particularly relevant in neurological disorders such as multiple sclerosis, where demyelination alters the lipid and protein composition [30]. Conventional histopathology provides valuable structural information, but it requires staining and does not directly reflect the underlying biochemical changes. By contrast, MIR imaging can deliver label-free molecular contrast, enabling a more direct assessment of tissue integrity and disease-related alterations. Beyond neurological applications, the ability to delineate biochemical differences has potential clinical value in oncology—for example, by improving the identification of tumor margins, where precise resection is critical. Furthermore, characterizing extracellular matrix components such as collagen and elastin may support the pathological assessment of vascular diseases, including aneurysms, where changes in tissue composition directly affect vessel stability. More broadly, capturing fine tissue architectures, such as myelinated axons, single cells, and microvascular networks, which typically measure less than 20 µm, requires a high spatial resolution [31,32,33]. Capturing these features supports the early detection of pathological changes and enables precise tissue characterization in combination with molecular imaging techniques. However, with the spatial resolution achieved in previous studies of the developed MIR system, such structures could not yet be reliably captured [9,16,17,24,25].

### 1.3. Limitations of Existing Mid-Infrared Scanning Approach

The optical system of the MIR system was simulated and optimized using the OpticCAD software (Opticad Corporation, Santa Fe, NM, USA, Version 10.043), yielding a theoretically possible spot size of 22 µm. To verify how closely this target was achieved in practice, the beam profile was subsequently measured using a slit-based method with the Ophir NanoScan 2s Pyro/9/5 system [9,24,25].

It was observed that variations in spot size were highly dependent on the beam profiler settings and positioning accuracy, which affected laser deviations. In-built lasers achieved the smallest spot size, ranging from 22.09 µm to 23.57 µm. To further enhance the resolution in mid-infrared scanning, a spatial rastering technique was applied, shifting the 20 µm spot in 5 µm steps. This method effectively increased the resolution from 20 µm to 10 µm. A line grating target (Thorlabs, Newton, NJ, USA, R1L3S6P) was placed in the scanning system’s object holder to assess the achievable resolution. This target includes 1.25 to 250 lines per mm across 18 sections, covering the range of the system’s best-possible resolution for validation. With the improved 10 µm spatial resolution, structures of up to 50 lines per mm were clearly visualized on the line grating target [16].

Due to the inability to visualize finer structures with the existing setup, a multimodal system was developed, incorporating an additional imaging path. This path leverages autofluorescence to provide not only a high spatial resolution but also additional chemical information.

### 1.4. Theoretical Background for Autofluorescent Targets

Fluorescence is a photophysical process in which a molecule absorbs light and transitions to an excited electronic state. Upon returning to the ground state, typically within nanoseconds, it emits a photon of lower energy and therefore a higher wavelength (Stokes shift) [34]. Autofluorescence refers to the intrinsic fluorescence of biological structures without the need for external dyes. It arises from endogenous fluorophores such as flavins (FAD) [35], aromatic amino acid-containing proteins, lipopigments, and structural proteins like collagen and elastin [36,37,38,39]. These molecules exhibit characteristic excitation and emission spectra that vary depending on their molecular environment. In biological tissues, extracellular matrix components, particularly collagen and elastin, often dominate the autofluorescence signal due to their high quantum yields. For example, flavins have excitation maxima at 360 nm and 450 nm, with emissions between 500 nm and 600 nm [35]. Lipopigments, which are associated with aging and disease, emit light at 450 nm and again around 600 nm. The intensity and spatial distribution of autofluorescence can provide insights into tissue compositions and metabolic states, making it a valuable tool for real-time, label-free imaging. Therefore, this approach is used as an additional autofluorescence-based optical path in a multimodal imaging system, designed to enhance the spatial resolution and diagnostic capabilities in the analysis of medical samples [40,41,42,43,44].

## 2. Materials and Methods

In this study, a multimodal optical system was developed to enable both fluorescence and mid-infrared (MIR) imaging within a unified platform. The setup features a motorized mirror that allows for rapid switching between the two modalities. This integrated configuration facilitates efficient data acquisition by combining high-resolution and high-contrast structural and chemical information from the same sample area.

The following section first details the mid-infrared (MIR) optical path, which serves as the core of the multimodal system, followed by descriptions of the overall setup, sample preparation, and data analysis algorithms.

### 2.1. Fast Mid-Infrared Scanning Path for High-Contrast Chemical Segmentation

**Figure 1:**
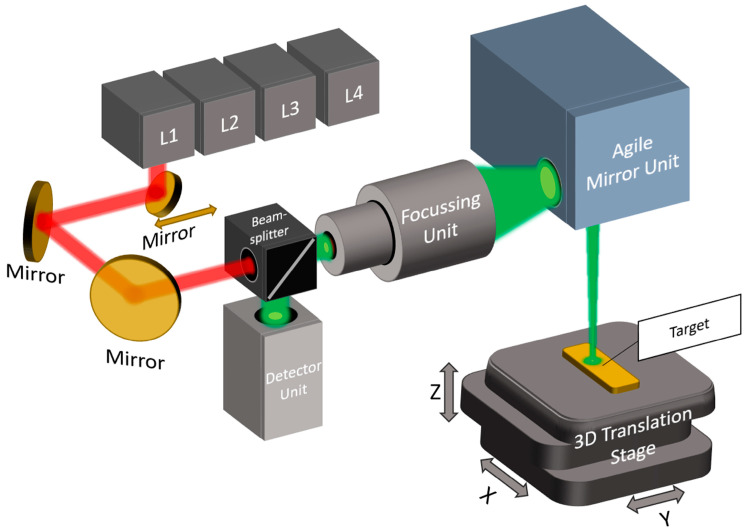
Schematic of the MIR scanning system with integrated lasers at 3704 cm^−1^ (L1), 3352 cm^−1^ (L2), 2928 cm^−1^ (L3), and 2792 cm^−1^ (L4). The laser paths are shown in red, while the confocal detection pathway is indicated in green.

A laser-based MIR scanner enables the fast, spatially resolved detection of lipids and proteins by targeting CH_2_ and NH vibrations. Using a flying spot method, it captures absorbance and scattering signals for the label-free chemical mapping of biological samples. The system uses four distributed feedback (DFB) lasers (Nanoplus Nanosystems and Technologies GmbH, Meiningen, Germany) emitting at specific wavenumbers: 2792 cm^−1^ (L4), 2928 cm^−1^ (L3), 3352 cm^−1^ (L2), and 3704 cm^−1^ (L1). These were chosen to target the valence vibrations of lipid and protein structures. L2 and L3 serve as target lasers for lipids and proteins, respectively, while L1 and L4 act as reference lasers, primarily for detecting scattering properties. This dual-laser approach allows the system to compensate for surface roughness and substrate interference through differential analysis using Beer–Lambert calculations, effectively isolating chemical absorption from background noise. This is because the reference lasers detect the scattering properties of the target [26,45,46,47]. The system’s MIR spectral coverage focuses on two key IR subregions: the functional group region and the fingerprint region. These spectral ranges contain strong absorption bands for molecular groups such as CH_2_ (2928 cm^−1^) [48] and NH (3200–3570 cm^−1^) [49,50,51]. By restricting measurements to four highly relevant wavenumbers instead of acquiring the full spectrum, the system achieves substantial increases in scanning speed. This results in acquisition rates up to 225 times faster than in conventional Fourier transform infrared (FTIR) imaging, without sacrificing chemical specificity [9]. The optical setup shown in Figure 1 features a confocal arrangement that guides collimated laser beams sequentially through a lens system optimized for minimal spot sizes. The laser light is guided via a mirror mounted on a motorized linear axis that selects and aligns each laser with the focusing optics. Confocal detection was performed using a high-sensitivity IR detector (VIGO Photonics S.A., Ożarów Mazowiecki, Poland; D* ≈ 4 × 10^−10^ cm Hz½ W^−1^), capable of sampling at ~2.7 MS/s. The system achieves spatial resolutions of 20 µm and can scan 1 cm^2^ areas in approximately 3 s per laser. Using the method from Section 1.3, a scan area of 1 cm^2^ can be acquired with a resolution of 10 µm in 12 s. The focused beam is deflected by agile mirror units to form a scan line, while a 3D translation stage moves the sample orthogonally to complete the scan field. Further, the system enables hydration correction by using reference lasers within and outside water absorption bands. Subtracting scattering signals (L1, L4) from absorption data corrects for surface irregularities, ensuring accurate chemical fingerprinting [9,16,17,24,25].

### 2.2. Imaging Path for High-Resolution Chemical Segmentation of Autofluorescent Targets

The fluorescence imaging system was specifically developed to detect the autofluorescence of various endogenous biomolecules, such as collagen, elastin, porphyrins, lipopigments, NADH, and flavins. These biomolecules exhibit characteristic excitation and emission spectra, which were selectively addressed by dedicated combinations of excitation light-emitting diodes (LEDs) and band-pass emission filters [14,35,52,53].

Fluorescence excitation was provided by LEDs mounted in a circular ring configuration around the sample. This arrangement ensures homogeneous illumination across the field of view. The illumination unit consists of multiple LEDs at four discrete wavelengths (355 nm, 395 nm, 435 nm, and 440 nm; XSL-355-5E-R6, LED395-01, LED435-12-30, and LED440-6-30, Roithner, Vienna, Austria), with three LEDs per wavelength, symmetrically arranged to ensure the homogeneous excitation of the sample. The emitted fluorescence passes through a 65 mm zoom (1-5x) lens (Canon, Tokyo, Japan) with a maximum aperture of f/2.8, which focuses the image before it reaches a motorized filter wheel. The filter wheel holds band-pass filters centered at 400 nm (10 nm bandwidth), 440 nm (10 nm), and 550 nm (40 nm) (FBH400-10, FB440-10, and FB550-40, Thorlabs, Newton, NJ, USA). These filters selectively transmit the emitted fluorescence from specific biomolecules while blocking excitation light and background signals. Image acquisition was performed by a high-resolution CCD camera (4499 × 3599 pixels) with a pixel size of 6 µm × 6 µm, enabling detailed spatial resolution and automated image capture (Atik 16200, Atik Cameras, Santa Iria de Azoia, Portugal). A gain factor of 0.6e-/ADU, as well as a dark current of >0.25 electrons/second at 0 °C, ensure low noise (typical readout noise: 9e-) and high image quality even with only small fluorescence signals. The exposure time was optimized individually for each targeted biomolecule based on its fluorescence intensity and spectral characteristics, ranging between 5 s and 50 s to ensure a sufficient signal-to-noise ratio without overexposure. Each target biomolecule was stimulated and detected using the following combinations of LEDs and emission filters.

- Collagen and Elastin: Excitation occurs in the 320–360 nm range, with fluorescence emission between 400 and 440 nm. Detection was performed using a 355 nm LED and two band-pass filters centered at 400 nm and 440 nm (FWHM: 10 nm) [14,52].
- Lipopigments: Excited between 400 and 500 nm with emission spanning 480–700 nm. A 440 nm LED was used in combination with a 550 nm band-pass filter (FWHM: 40 nm) for detection [14,52].
- Flavins: Excitation peaks are located at 350–370 nm and 440–450 nm, with emission from 500 to 600 nm. Both 355 nm and 440 nm LEDs were used for excitation. Emission was detected using the 550 nm filter (FWHM: 40 nm) [14,35,52,53].

**Figure 2:**
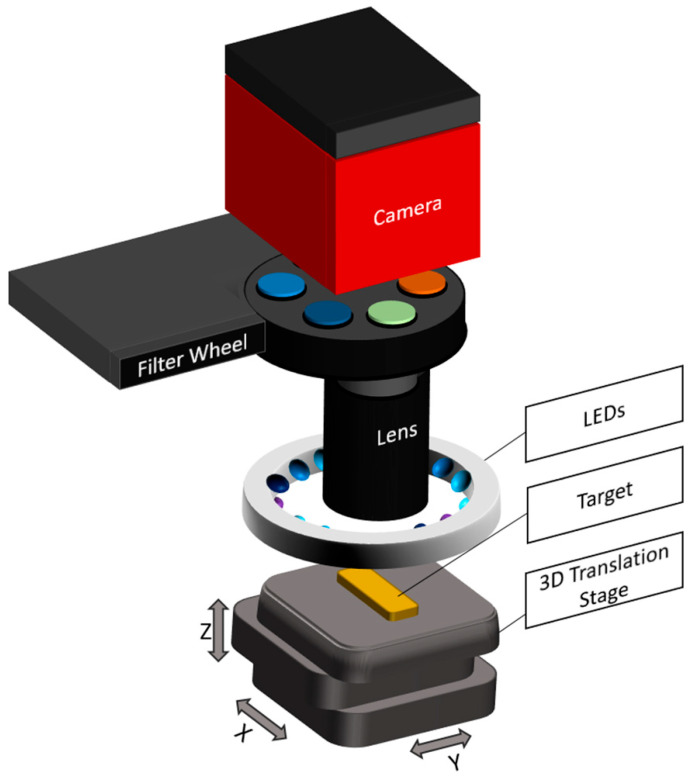
Schematic of the autofluorescence imaging system with integrated filters at 400 nm, 440 nm, and 550 nm and LEDs with 355 nm, 395 nm, 435 nm, and 440 nm.

To ensure spectral fidelity and reliable system performance, all excitation and detection components were characterized using a spectrometer (MCS601 UV-NIR C, Zeiss, Oberkochen, Germany). The LED output was measured at the sample plane to confirm both wavelength accuracy and irradiance. Transmitted light through each emission filter was measured at the camera position using a fiber coupled in the optical pathway of the system (Optical Fiber Jacket 1000 µm, Edmund Optics, Barrington, NJ, USA), verifying that each filter passed only the designated spectral band to the sensor. The complete optical assembly, shown in Figure 2, is housed in a custom metal enclosure coated internally with a highly light-absorbing material. This design minimizes stray light and internal reflections, ensuring high image contrast, an optimized signal-to-noise ratio, and effective spectral separation for the sensitive detection of biomolecular autofluorescence. Due to the substantial spectral overlap of the fluorescence emission bands among the targeted biomolecules, the quantitative analysis of specific substance concentrations is not feasible. The system is therefore limited to producing spatially resolved autofluorescence images that reflect relative chemical differences.

### 2.3. Multimodal Optical Path Configuration for Mid-Infrared and Autofluorescence Measurements

**Figure 3:**
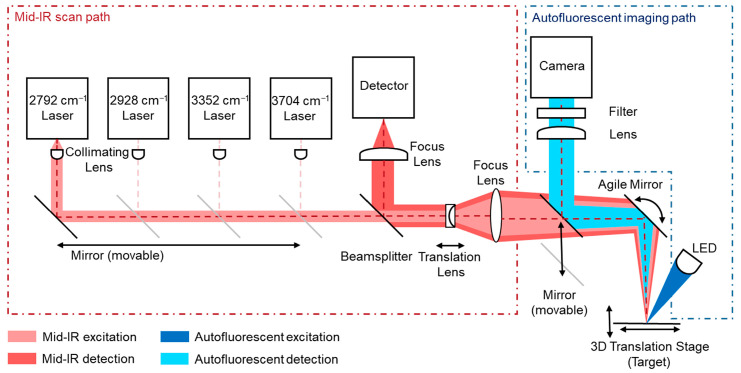
Schematic overview of the multimodal imaging approach. A movable mirror is used to align the optical paths of both systems into a single pathway.

The multimodal imaging system combines the mid-infrared laser scanning path and the fluorescence imaging path within a shared optical architecture. A motorized mirror was used to dynamically switch the optical path between the MIR detector and the fluorescence camera, allowing both modalities to operate within the same optical axis. The described mirror directs the imaging system along the optical path of the overall setup into the agile mirror unit of the MIR system, as shown in Figure 3. This co-alignment ensures that both datasets are inherently registered, without requiring external alignment procedures or separate optical systems. Functionally, the MIR path offers high chemical contrast by detecting molecular absorption and scattering at selected IR wavenumbers (targeting CH_2_ and NH vibrations), but with a limited spatial resolution due to diffraction constraints at MIR wavelengths. In contrast, the fluorescence path captures high-resolution images of autofluorescent biomolecules using shorter-wavelength excitation and emission light, enabling fine structural detail through high-resolution CCD imaging. The agile mirror unit remains still while imaging autofluorescence. By sharing the same optical path, the system allows fluorescence images to serve as spatially precise reference maps for the MIR data. Structural features resolved in the fluorescence modality were used to guide the interpretation, segmentation, and potential super-resolution enhancement of the MIR chemical maps. This integrated design provides both molecular specificity (via MIR) and a subcellular resolution (via fluorescence), resulting in a powerful tool for the comprehensive analysis of biological and material samples. The shared-path architecture also reduces the system complexity, alignment errors, and the overall footprint, enabling faster switching and precise multimodal overlay.

**Figure 4:**
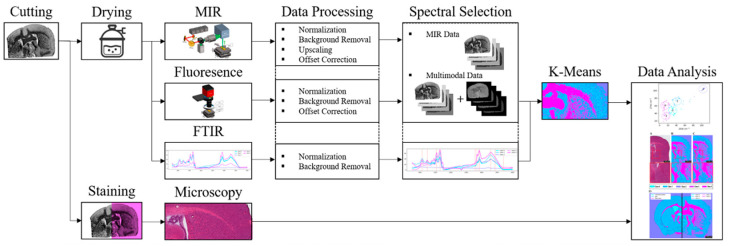
Workflow of the experimental procedure, including sample preparation, imaging with MIR and fluorescence, imaging with FTIR and H&E staining reference methods, raw data processing, K-means clustering (k = 5), and subsequent result analysis.

Using the described setup in combination with FTIR and H&E as reference methods, the prepared samples were imaged. The resulting raw data were subsequently processed and clustered using K-means (k = 5), followed by result analysis. The complete workflow is summarized in the flowchart presented in Figure 4.

### 2.4. Sample Preparation

The samples were obtained from C57BL/6JRj mice, aged 63 weeks. All animal procedures were conducted in accordance with ethical regulations and approved under permit number I-24/01. For the purposes of this study, three pairs were prepared to capture the coronal brain section at level 58, corresponding approximately to Bregma −1.0 mm, as defined by the Allen Brain Atlas for the mouse brain [54]. Brain samples were frozen free-floating on liquid nitrogen. When frozen, samples were stored at −80 °C until use. Tissue sectioning (10 µm thickness) of brain tissue was performed using a CM1860UV cryostat (Leica Biosystems GmbH, Nußloch, Germany) with a chamber temperature of −15 °C. One tissue section of each pair was mounted on a gold-coated glass slide (MN10-AU8632, Science Services GmbH, Munich, Germany) and analyzed spectrally using FTIR imaging, followed by multispectral evaluation. The second section was placed on a SuperFrost Plus Adhesion glass slide (VWR International GmbH, Darmstadt, Germany) and underwent hematoxylin and eosin (H&E) staining to enhance the visibility of the tissue’s morphological structures. Prior to spectral measurement, the sample was dried in a desiccator for 10 min to minimize the water content, which was crucial to prevent the O–H vibrational bands from overlapping with the C–H and N–H stretching vibrations. Such overlap would impede the accurate measurement of the C–H and N–H bands in the functional group region. For examination with the MIR scanner, the recorded tissue was positioned in the sample holder of the instrument. The sample was automatically transferred into the measurement range of the device. After the precise alignment of the images, the sample was sequentially scanned using all four laser wavelengths, followed by fluorescent imaging. The resulting measurement images were subsequently utilized for the further analysis of the tissue section [9].

For the second tissue sample, hematoxylin and eosin (H&E) staining was performed as described elsewhere [PMID: 37345020] using 2 min hemalaun (Mayer’s hemalaun solution, HX14931949, Sigma-Aldrich, Merck KGaA, Darmstadt, Germany), 3 min tap water, 3 dips in dH2O, 1 min acidic alcohol (350 mL ethanol + 150 mL H2O + 1.5 mL HCl) (ethanol absolute EMPLURA^®^, 818760, Merck KGaA, Germany) (HCL Tritripur, 1.09057.1000, Merck KGaA, Germany), 3 dips in dH2O, 2 min blueing solution (2 g NaHCO3 + 20 g MgSO4 in 1 L H2O) (NaHCO3, 801K00801129, Merck KGaA, Germany) (MgSO4, 7154.1000, VWR Chemicals, Darmstadt, Germany), 3 dips in dH2O, 2 min acidified eosin (eosin Y-solution 0.5% aqueous, 1.09844.1000, Merck KGaA, Germany with 0.25% 1M HCl), 3 dips in dH2O, 1 min 80% ethanol, 2 min 96% ethanol, twice 1 min 100% ethanol, and 2 min xylene (Thermo Fisher Scientific, Waltham, MA, USA) and coverslipped with eukitt (03989-100 ML, Sigma-Aldrich, Merck KGaA, Darmstadt, Germany) and a glass cover slide (ECN 631-1575, VWR Chemicals, Germany). The slide was then imaged with a Leica Aperio Scanner (20x) (CS2, Leica Biosystems GmbH, Nußloch, Germany) and viewed in AperioImageScape (Leica Biosystems GmbH, Germany) [9].

### 2.5. Measurement Conditions

The FTIR measurement data presented in this study were obtained using the Perkin Elmer Spotlight 400 FTIR imaging system (Perkin Elmer Inc., Shelton, CT, USA). For each experiment, spectra were collected over the wavenumber range of 4000 to 750 cm^−1^, with a spectral resolution of 8 cm^−1^. Measurements were conducted in reflection mode, chosen for its similarity to the scanning method employed by the MIR scanner. To minimize background noise, each measurement point was averaged over two accumulations. The scanning speed was maintained at 2.2 cm/s. Prior to data acquisition, the FTIR detector was cooled using liquid nitrogen.

For the multispectral analysis, the samples were positioned in the sample holder of the measurement device. After the precise alignment of the images, the sample was sequentially scanned using all four laser wavelengths. Scans in the mid-infrared range were conducted at wavenumbers of 2790 cm^−1^, 2926 cm^−1^, 3350 cm^−1^, and 3700 cm^−1^. The resulting resolution was 10 µm, with a scan speed of 12 s/cm^2^. The detector operated with sensitivity of approximately 4 × 10^−10^ cmHz^1^/^2^/W and a bandwidth of up to 2.7 GHz. Subsequently, fluorescence images were captured with detection filtering at 400 nm (10 nm bandwidth), 440 nm (10 nm bandwidth), 460 nm (10 nm bandwidth), 550 nm (40 nm bandwidth), and 633 nm (3 nm bandwidth). The sample was illuminated at wavelengths of 355 nm, 365 nm, 395 nm, 435 nm, and 440 nm, with exposure times ranging from 5 to 50 s. Filter and LED combinations were used to image collagen, elastin, NADH, flavin, lipopigments, and porphyrin according to Section 2.2. The exposure times were optimized to ensure a high-contrast signal on the camera. Using a magnification objective, structures smaller than 3 µm were visualized.

### 2.6. Data Processing and Applied Algorithms

Raw data acquired from Fourier transform infrared (FTIR), mid-infrared (MIR), and fluorescence imaging techniques were initially recorded as light intensities within the range of 0 to 65,535 counts. These values were normalized to a scale of 0 to 1. The FTIR system utilized an automatic background subtraction feature. In contrast, the custom-built MIR system employed the gold-coated slide itself, representing the maximum signal, and an intrinsic reference within the slide. This system measures both scattering and absorbance at multiple wavelengths, leveraging the local scattering properties of the sample or substrate as an intrinsic reference. By using scattering as a reference, the system compensates for suboptimal background conditions, thereby ensuring accurate spectral detection even on rough or uneven surfaces [26,45,46,47]. To address potential issues arising from small pixel or image offsets in the MIR recordings, the system setup was calibrated to a movable microscope stage, ensuring precise alignment during imaging. To improve the spatial resolution of the MIR images, upscaling was applied with HSI-Wizard DataCube.resize (LINEAR_CUBIC) [55]. Upscaling of the MIR data to match the 4 μm autofluorescence resolution may introduce interpolation artifacts, which could slightly affect the spatial accuracy of the chemical maps. This limitation should be considered when interpreting fine spatial features in the data. The data obtained from MIR and fluorescence imaging were subjected to a clustering analysis using the K-means algorithm and compared to K-means clustering with FTIR [56]. K-means clustering was employed as it enables the clear and computationally efficient partitioning of spectral data, which can be directly mapped to spatial regions. In comparison, principal component analysis (PCA) primarily serves dimensionality reduction without producing discrete clusters, while hierarchical clustering is less suited for large datasets owing to its higher computational demands. To correct for minor positional offsets inherent to the measurement system, the images were spatially registered using the wizard._core.datacube_ops.register_layers_best function. The K-means clustering method, an unsupervised machine learning technique, was applied to partition the high-dimensional data into distinct groups, thereby facilitating their visualization and interpretation. In this study, both MIR and fluorescence data were integrated into the clustering process. The number of clusters and their corresponding colors were adjusted to ensure that the resulting classification closely aligned with known tissue structures, such as those found in brain atlases or histological staining patterns (e.g., H&E staining). This was achieved using k = 5 clusters, which provided the most meaningful representation of the tissue types and structures within the mouse brain samples. The choice of five clusters balances anatomical interpretability and clustering quality: it captures the four main compartments—background, ventricular space, gray matter, and white matter—while also allowing for the inclusion of subtle subregions or outliers. This selection was further supported by evaluation of the Davies–Bouldin index, which indicated optimal or near-optimal cluster compactness and separation at k = 5 (with 0.859 for a 6.25 µm resolution for FTIR using 4 wavenumbers), ensuring a close match to the expected anatomical and histological features [56,57,58].

Several factors, including the tissue composition, cell medium, and manual markings, influenced the measurements, thus necessitating the use of multiple clusters to ensure the robustness of the data analysis. As the number of clusters increased, assigning accurate chemical interpretations to the resulting sub-areas became more complex. Therefore, the analysis was performed with k = 5, as fewer clusters did not yield informative distinctions. The clustering procedure involved 10 iterations of the enhanced center initialization method [55].

The Dice coefficient and normalized mutual information (NMI) were excluded due to their sensitivity to minor misalignments and resolution differences after resampling and cropping. Instead, the adjusted Rand index (ARI) was used, as it provides a robust, label-independent measure of the clustering agreement across datasets with differing spatial scales. To quantitatively assess the agreement between different segmentations, all images were first aligned and resampled to a common spatial resolution and field of view. Segmentations were then cropped to ensure identical dimensions, and corresponding cluster labels were compared using the adjusted Rand index (ARI). This procedure allows for a fair comparison across datasets differing in spatial resolution or spectral content, while accounting for chance agreement and label permutations. The resulting ARI values provide a robust measure of the structural and chemical consistency between segmentations.

## 3. Results

Mid-infrared (MIR) imaging was conducted on coronal brain sections (level 58 according to the Allen Mouse Brain Atlas, approximately Bregma +1.0 mm) to differentiate major tissue compartments like white matter and gray matter based on their distinct chemical compositions. Laser excitation at 3352 cm^−1^ targeted CH_2_ stretching vibrations characteristic of lipid chains, whereas excitation at 2928 cm^−1^ probed NH stretching modes associated with protein content. White matter regions, such as the corpus callosum and fornix, are highly enriched in lipids due to compact myelin sheaths, while gray matter contains a higher density of neuronal and glial cell bodies, characterized by an elevated protein fraction and comparatively lower lipid content. Given these molecular differences, it was expected that MIR absorbance at the selected wavenumbers would provide sufficient chemical contrast for label-free tissue classification.

### 3.1. Resolution Effects on Tissue Clustering Observed with Conventional Methods

First, Fourier transform infrared (FTIR) imaging of the coronal brain section was performed to obtain high-resolution chemical maps. Subsequently, a K-means cluster analysis was carried out using the ten diagnostically relevant absorption bands identified from the FTIR dataset [16,59,60,61]. In addition to the four absorption bands employed in the mid-infrared scanner, the following six bands were included: 3450 cm^−1^ (OH), 2878 cm^−1^ (CH), 1655 cm^−1^ (amide I), 1550 cm^−1^ (amide II), 1320 cm^−1^ (amide III), and 1159 cm^−1^ (C–O) [16,59,60,61]. For comparison, an additional cluster analysis was performed using only the four wavelengths implemented in the prototype of the mid-infrared scanning system. The cluster results from both approaches were evaluated to determine whether the chemical segmentation corresponded to the expected anatomical regions and how their quality differed. Finally, the mean spectra for each cluster were calculated to highlight the expected absorption differences in the selected bands.

**Figure 5:**
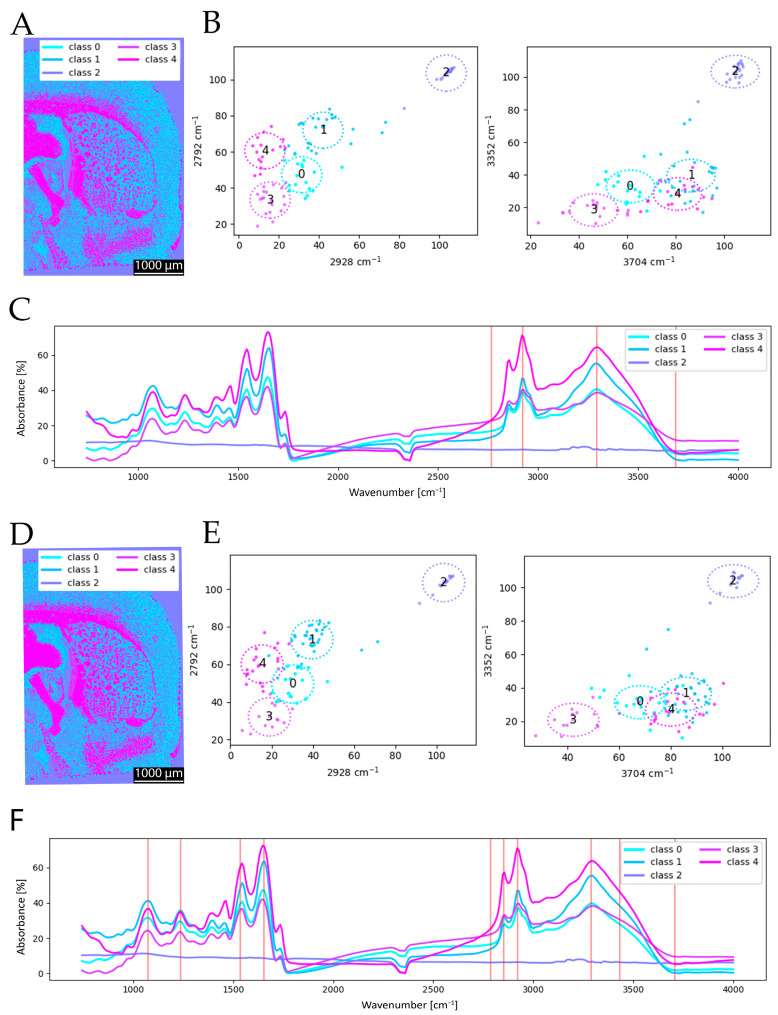
Comparison of spectral clustering using 4 wavenumbers (**A**–**C**) versus 10 wavenumbers (**D**–**F**) revealed comparable clustering results. Panels (**A**,**D**) show the resulting clustered images, (**B**,**E**) present scatterplots illustrating the clustered groups of the overlapping 4 wavenumbers, and (**C**,**F**) display the mean spectra for each clustered class. The reduction in spectral information does not substantially diminish the quality, provided that the most relevant peaks are retained.

As shown in Figure 5, the clustering results obtained with 4 and 10 selected wavenumbers exhibit only minor differences. In the spectral data, the mean spectrum of cluster group 4 displays the expected high absorption at 3352 cm^−1^ (Figure 5C). For cluster groups 0 and 1, which predominantly cover gray matter, protein absorption is stronger than lipid absorption. Furthermore, cluster group 0 shows even higher protein absorption than group 1, manifesting in a reticular pattern. This can be anatomically explained by extremely protein-rich fine structures such as dendritic and axonal fiber bundles or the molecular layer (Lamina I). Based on the mean spectra, it becomes evident that additional peaks can positively influence clustering, as observed in the analysis using 10 wavenumbers. It has to be noted that the mean spectra show no significant difference except for a slight increase at 1159 cm^−1^ (C–O) for the clustering with 10 wavenumbers (compare Figure 5C to Figure 5F). Nevertheless, the overall clustering results remain highly similar, and the added value of the extended spectral set has only a minor impact on the final segmentation. The scatterplots (Figure 5B,E) also underline this, as the locations of each cluster group have comparable positions. This observation is consistent with findings from a previous study, which also reported only marginal qualitative improvements when increasing the number of spectral features [16].

This visual impression is further supported by ARI = 0.727, quantitatively confirming the strong agreement between both segmentations despite the differing spectral inputs.

**Figure 6:**
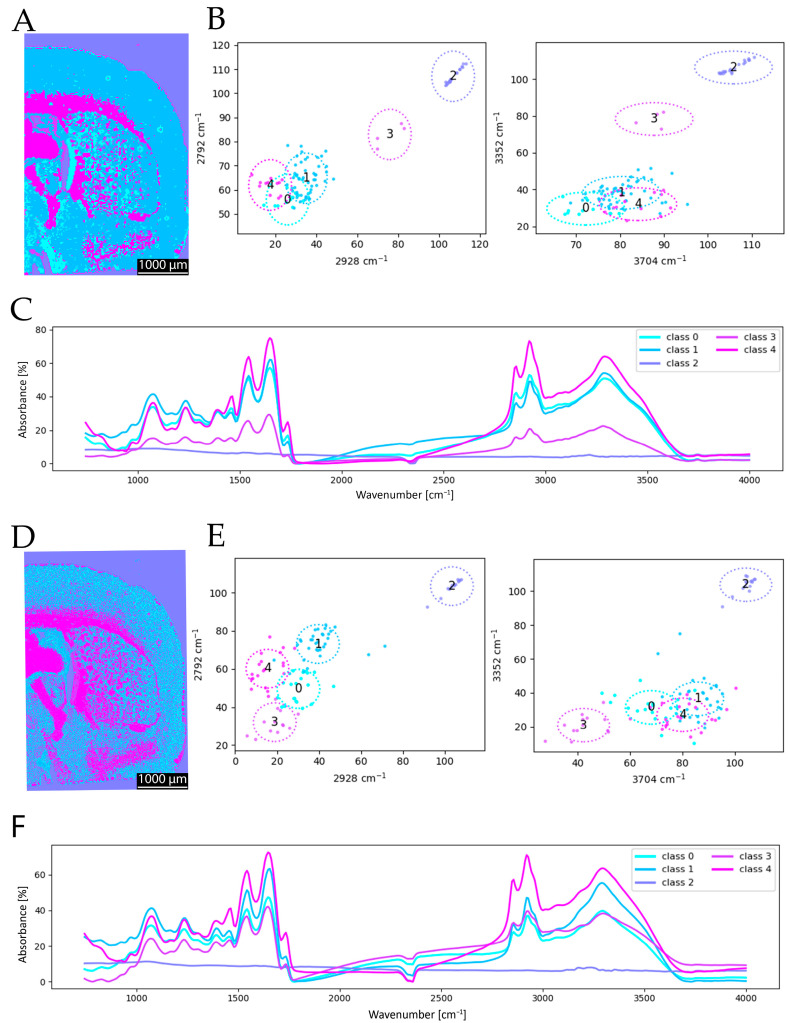
Comparison of spectral clustering at different spatial resolutions, 25 µm (**A**–**C**) versus 6.25 µm (**D**–**F**). Panels (**A**,**D**) show the resulting clustered images, (**B**,**E**) present scatterplots illustrating the clustered groups of overlapping wavenumbers, and (**C**,**F**) display the mean spectra for each clustered class. The reduction in special resolution substantially diminishes the quality, manifesting also in the spectral data, especially for cluster 3.

Following the initial cluster analysis, a resolution comparison was conducted based on the FTIR-derived segmentation maps. For this purpose, the coronal brain section was imaged using FTIR at spatial resolutions of 6.25 µm and 25 µm. The resulting cluster maps (Figure 6) clearly illustrate the advantages of a higher spatial resolution. In particular, fine anatomical structures and boundary regions in the segmentation are more sharply delineated at 6.25 µm (Figure 6A compared to Figure 6D). Additional structural details are discernible in the high-resolution clustering that are not captured at a lower resolution, thereby enhancing the overall quality and anatomical accuracy of the segmentation. The 6.25 µm resolution provides more detailed and accurate clustering, whereas the 25 µm clustering lacks sufficient detail to clearly resolve specific structures. Consequently, a general increase in spatial resolution is desirable for improved anatomical discrimination. Observing the mean spectra for the two resolutions, the differences are more noticeable than in the comparison with a different number of included wavenumbers. This underlines the above-mentioned arguments. While, for 6.25 µm, classes 0 and 3 are spectrally very close to each other in the mean spectra (Figure 6F), for 25 µm, class 3 is very low in absorbance in general (Figure 6C). Further, classes 0 and 1 are very close for 25 µm. The shift in class 3 is also visible in the scatterplots, as the location of class 3 changes in comparison (Figure 6B,E). This observation is supported by the adjusted Rand index (ARI = 0.640), which indicates a lower level of agreement compared to the spectral resolution study, highlighting that the spatial resolution has a stronger influence on the segmentation outcome than a reduction in spectral information.

### 3.2. Photometric Spectral Clustering and Anatomical Correspondence of MIR Scans

After the preparatory analysis using the commercially available FTIR reference method, the resolution effect was also examined with the MIR prototype. Scans acquired at a pixel size of 20 µm were compared with those obtained at a finer raster of 5 µm, enabling the visualization of smaller structures (10 µm). The higher-resolution data were subsequently resampled to match the native camera resolution (4 µm), following the data processing approach described in Section 2.6. The results revealed similar resolution-dependent benefits as observed for the FTIR analysis: fine structural details and sharper segmentation boundaries were more accurately represented at a higher resolution, enhancing the anatomical fidelity of the chemical maps.

**Figure 7:**
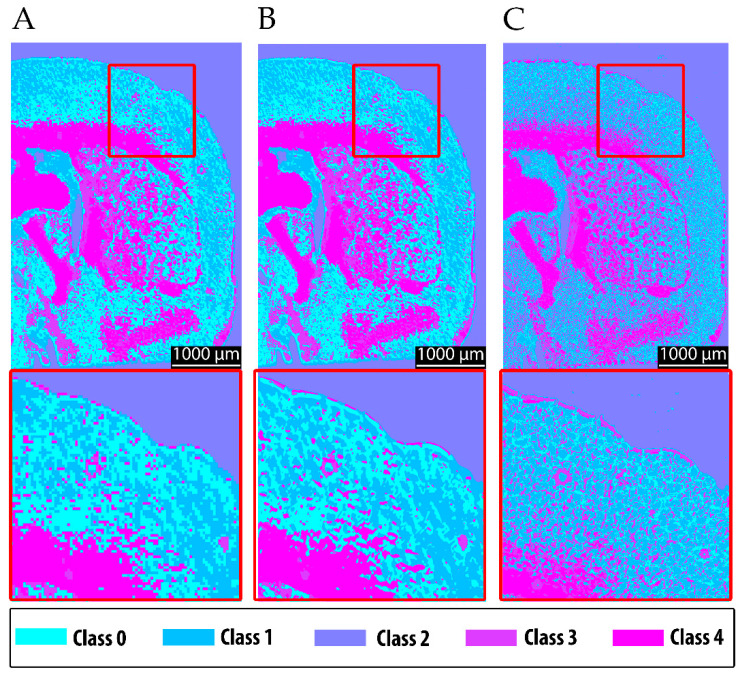
Comparison of clustered images from different imaging modalities and resolutions with magnification of 2.5. Panel (**A**) shows the MIR scan at 20 µm, (**B**) shows the 10 µm MIR data upscaled to 4 µm, and (**C**) presents the clustered image from a 6.25 µm FTIR scan. Chemical rationale translated successfully into spatial segmentation, with clustering patterns aligning with both the anatomical structure and underlying molecular composition. The 6.25 µm FTIR clustering shows finer structures, even compared to the scaled-up MIR scan.

The resulting segmentation produced spatially coherent clusters that corresponded well to known neuroanatomical features and reflected the expected chemical contrast. White matter structures—notably the corpus callosum, the fornix system, and epithalamic fiber tracts—were grouped in pink, consistent with their strong lipid-associated absorbance at 3352 cm^−1^ (Figure 7). Gray matter regions, including the caudoputamen, pallidum, and olfactory areas, formed chemically distinct clusters based on their moderate absorbance profiles and mixed lipid and protein compositions. These findings confirm that the predefined chemical rationale translated successfully into spatial segmentation, with clustering patterns aligning with both the anatomical structure and underlying molecular composition. The ability to differentiate between gray matter, white matter, and ventricular regions based solely on MIR absorption supports the robustness and specificity of the selected method.

It is evident that the more detailed raw data acquired at 10 µm, when upscaled to the camera resolution of 4 µm, improve the clustering performance, particularly for fine structural features, in comparison to the 20 µm resolution (compare Figure 7A to Figure 7B). Nevertheless, fine structures are still delineated with higher quality using the 6.25 µm FTIR data (Figure 7C). These findings motivated the multimodal approach, in which 4 µm raw data were incorporated into the chemical clustering of both fine-structured and upscaled MIR scans.

### 3.3. Multimodal Approach for Chemical Clustering of Tissue

In the next phase of this study, autofluorescence imaging was integrated to enhance tissue discrimination further. By targeting endogenous fluorophores related to cellular metabolism and extracellular structures, this modality was expected to provide complementary contrast and refine the classification of complex tissue regions beyond what can be resolved through infrared absorption alone. Different fluorophores are known to correlate with distinct tissue types. White matter is anticipated to show enhanced signals from FAD due to its presence in the mitochondria of metabolically active axons and glial cells, as well as from collagen and elastin, which are prevalent in perivascular and structural compartments. In contrast, gray matter, rich in neuronal and glial cell bodies, is expected to exhibit stronger autofluorescence from lipopigments such as lipofuscin and porphyrins, associated with neuronal aging and heme metabolism. In addition to improving the chemical specificity, the high spatial resolution of fluorescence microscopy will enable the visualization of finer anatomical details and microstructural features that remain unresolved in mid-infrared maps, thereby providing a more nuanced and spatially detailed view of tissue organization.

This integration of autofluorescence data significantly improved the clustering performance. The expected contrast between gray and white matter was successfully reinforced through the combined analysis, enabling the much clearer separation of these major tissue compartments than was possible using MIR data alone.

**Figure 8:**
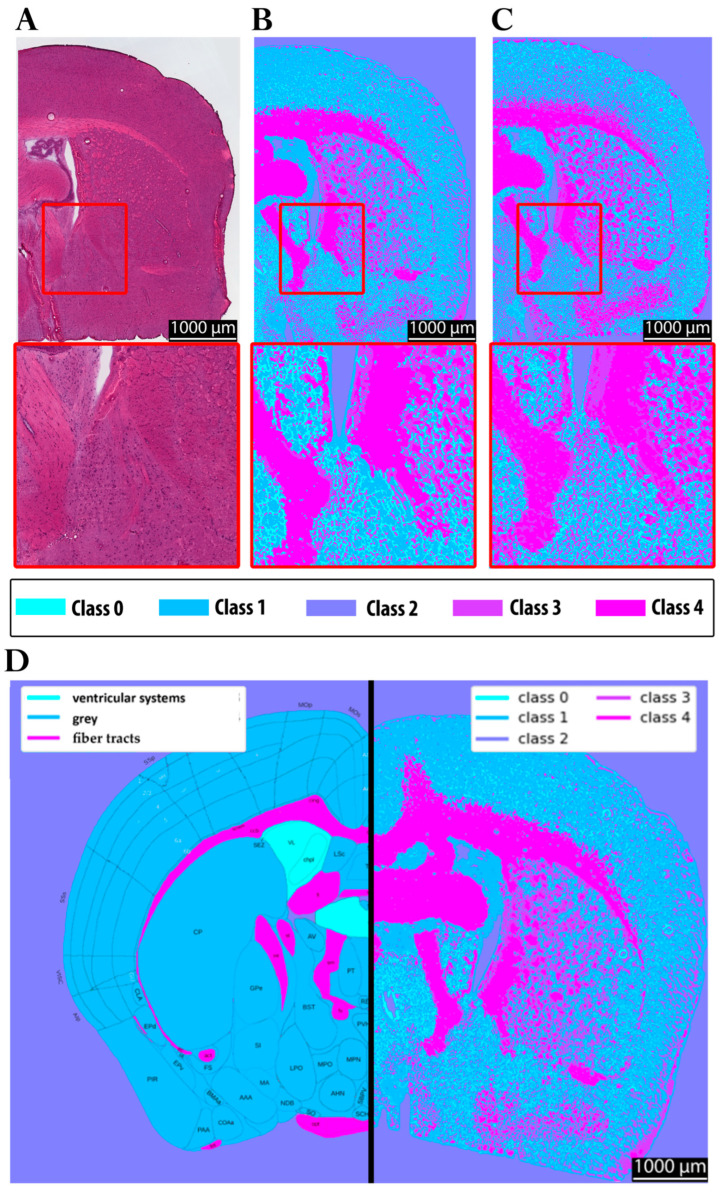
Multimodal imaging and clustering analysis with magnification of 2.5. (**A**) H&E-stained brain section. (**B**) Multimodal clustering combining 4 µm upscaled 10 µm MIR scan with 4 µm autofluorescence. (**C**) FTIR scan of the same region at 6.25 µm. (**D**) Representation of the corresponding section plane (58) from the Allen Brain Atlas (**left**) and segmentation using the multimodal approach, with the area shown in (**B**) (**right**). The figure highlights how comparable the multimodal approach (**B**) is to FTIR (**C**), while simultaneously illustrating the clear contrast with H&E staining (**A**).

In Figure 8, panel A shows the hematoxylin and eosin (H&E) staining of the investigated brain section. Panel B depicts the corresponding clustering results obtained using the multimodal approach, highlighting the chemical contrast within the tissue. Panel C shows the clustered FTIR data at 6.25 µm for reference. For anatomical reference, panel D displays section 58 of the Allen Brain Atlas, color-coded to distinguish gray matter, white matter, and vascular structures, enabling a comparison with the experimental data. Panel E presents a full-brain clustering map acquired with the multimodal approach. Such a complete dataset could not be obtained with FTIR imaging due to the extensive acquisition time and data volume. In fact, only half of the tissue section was measured with FTIR in a scan lasting approximately eight hours, while the multimodal approach allowed the visualization of the entire section within a feasible timeframe (<5 min).

The clustering results (panels B, C, and E) reveal structural differences within the tissue more clearly than the H&E staining, despite the latter’s ability to highlight specific histological features such as cell nuclei, which are not resolved in the chemical clustering. Nevertheless, the clustering approach successfully differentiates regions corresponding to distinct tissue types, as also indicated by the color variations in the H&E staining. Moreover, when compared to the Allen Brain Atlas (panel D), both the multimodal and FTIR-based clustering approaches yield gray–white matter distinctions consistent with the anatomical reference, underscoring their potential for label-free tissue characterization.

**Figure 9:**
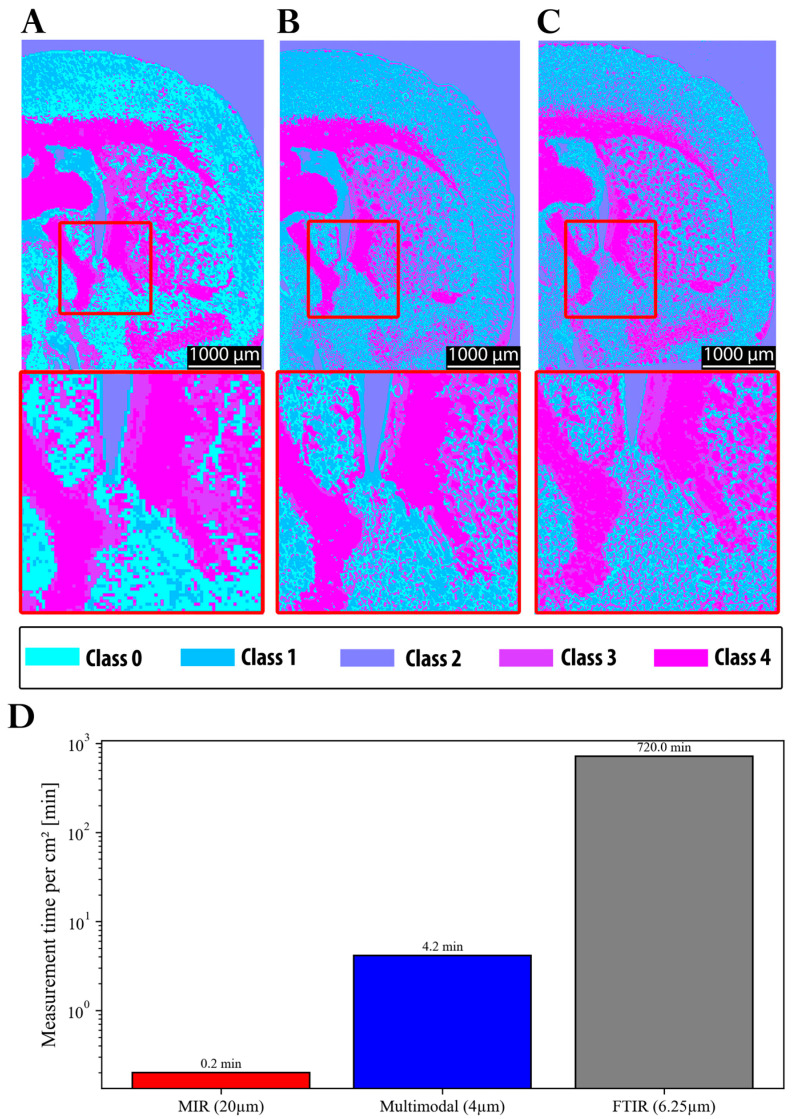
Comparison of clustering results across imaging approaches with magnification of 2.5. (**A**) Clustering obtained with the MIR prototype prior to the multimodal approach used in this study. (**B**) Clustering from the current study using 10 µm MIR scans upscaled to 4 µm and combined with 4 µm autofluorescence images. (**C**) Clustering from a 6.25 µm FTIR scan of the same region. (**D**) Comparison of acquisition times for the three imaging approaches.

In summary, the main progress achieved in this study is illustrated in Figure 9. The initial resolution of the MIR system was insufficient to reliably capture fine structural details. Moreover, when clustering was performed using MIR data alone, class 0 was predominantly assigned to gray matter regions (Figure 9A). In contrast, both FTIR (Figure 9C) and the multimodal approach (Figure 9B) assigned these regions primarily to class 1, with class 0 instead representing finer structures in the gray matter region. These discrepancies can be attributed to the impact of the spatial resolution on the clustering outcomes, as well as to differences in the average spectra of the segmented classes (see Section 3.1). Notably, the clustering results obtained from FTIR and the multimodal approach were highly consistent in terms of segmentation quality. This is also reflected by the ARI, which shows markedly higher agreement between FTIR and multimodal segmentation (ARI = 0.787) than between FTIR and the MIR-upscaled data (ARI = 0.575), confirming that the multimodal approach provides superior structural consistency. Furthermore, Figure 9D demonstrates the significant reduction in acquisition time achieved by the MIR prototype (0.2 min) and the multimodal approach (4.2 min), compared to FTIR (720 min). This corresponds to a speed increase of several orders of magnitude for MIR (~factor 3600), albeit with a loss of structural fidelity, and a speed improvement by a factor of approximately 171 for the multimodal approach, while maintaining comparable quality to FTIR.

## 4. Discussion

The present findings demonstrate that both MIR- and FTIR-based imaging approaches enable the reliable clustering of chemically and structurally distinct tissue regions, confirming the suitability of spectral clustering across modalities. Importantly, reducing spectral information does not necessarily lower the clustering performance, provided that the remaining spectral bands capture the most relevant molecular signatures. This is supported by the comparison between 6.25 µm FTIR scans with 10 and 4 spectral bands, which yielded a high ARI of 0.778, indicating that the segmentation remains highly consistent despite the reduced spectral dimensionality. The spatial resolution emerged as the dominant factor shaping anatomical precision and chemical specificity. For example, the ARI between 6.25 µm and 25 µm FTIR scans with 10 spectral bands was lower (ARI = 0.640), reflecting the stronger impact of a reduced spatial resolution on the clustering quality. A higher spatial resolution consistently improved the delineation of structural boundaries and enhanced the segmentation accuracy, allowing subregions to be assigned more reliably to distinct chemical classes. These observations align with previous work on bacterial samples, where a higher spatial resolution produced more detailed and accurate clustering. The current results extend this principle to complex brain tissue, demonstrating that the benefits of improved resolutions are not confined to microbial systems but also hold for highly heterogeneous anatomical structures.

Despite these advances, MIR imaging at standard resolutions still produced relatively coarse segmentation, with partial overlap between tissue compartments, as shown in Figure 9A. Boundary definition and chemical specificity were substantially improved in the 6.25 µm FTIR scans, which combined high spectral richness with a superior spatial resolution (Figure 9C). By comparison, MIR imaging alone remained limited in resolving fine structures, a constraint directly attributable to its reduced spatial resolution. This difference in segmentation quality is also reflected quantitatively by the adjusted Rand index (ARI): the comparison between the 6.25 µm FTIR and MIR-upscaled data yields an ARI of 0.575, whereas the comparison between the 6.25 µm FTIR and the multimodal approach reaches a higher ARI of 0.787, confirming that the multimodal method achieves superior structural agreement.

The biological relevance of these differences becomes clear when comparing white and gray matter. Regions of white matter, such as the corpus callosum and the fornix, which are rich in lipids from densely packed myelin, could be easily distinguished from gray matter, which has a higher density of protein-rich neuronal and glial cell bodies. However, the accurate imaging of structures such as these, which typically are of a size smaller than 20 µm [62,63,64], requires image resolutions beyond those achievable with coarse MIR scans. Integrating high-resolution autofluorescence imaging into the workflow eliminated this limitation, enabling multimodal clustering that combined chemical specificity with improved structural accuracy (Figure 9B).

Crucially, these gains in resolution must be balanced against the acquisition time. A 20 µm MIR scan, for instance, required only 0.2 min but produced segmentation that was too coarse for detailed anatomical analysis. In contrast, the multimodal approach combining 10 µm MIR scans upscaled to 4 µm with 4 µm autofluorescence data achieved clustering fidelity comparable to that of 6.25 µm FTIR scans in just 4.2 min. Direct FTIR imaging at 6.25 µm required 720 min to reach a similar quality level, making it impractical for large-scale or time-sensitive studies. Quantitatively, the multimodal workflow was ~171 times faster than FTIR, while retaining structural detail far superior to coarse MIR scans (Figure 9D).

From an application perspective, the trade-off between the resolution and acquisition speed is therefore far less limiting in the multimodal MIR setup than in conventional FTIR imaging. For contexts requiring fine structural differentiation, such as mapping biofilms or delineating anatomical subregions, the resolution gains clearly outweigh the moderate increase in acquisition time. Moreover, the multimodal approach substantially outperforms the previous MIR prototype, delivering improved chemical specificity and spatial detail while keeping scan times within practical limits. With a time saving of approximately 715 min/cm^2^ compared to conventional high-resolution FTIR, the method not only enables rapid intraoperative analyses but also facilitates high-throughput studies, large-area mapping, and near-real-time experimental feedback, greatly enhancing its practical applicability.

Comparison with conventional H&E staining further highlights the complementary strengths of these methods. While H&E offers unrivaled cellular detail and well-established diagnostic utility, it requires labor-intensive sample preparation and prolonged imaging times. The multimodal approach, by contrast, acquires label-free chemical maps of entire tissue sections within minutes, revealing structural and chemical information that is not accessible through H&E alone. Both methods showed strong concordance with anatomical references such as the Allen Brain Atlas, underscoring their combined value for tissue analysis. Overall, the integration of high-resolution MIR imaging with autofluorescence data provides a practical and time-efficient solution for chemical tissue characterization. This approach bridges the gap between speed, resolution, and analytical accuracy, offering a scalable method for research and potential clinical applications, including rapid tumor margin delineation or intraoperative tissue assessment. It should be noted that this study is a preliminary feasibility assessment limited to normal mouse brain and skin tissues. Future work should investigate the inclusion of additional MIR bands within the fingerprint region to fully exploit the potential of the multimodal approach. This is particularly relevant for the classification of heterogeneous or pathological tissues, where it remains to be evaluated whether four discrete wavenumbers are sufficient to achieve clear differentiation. Although this study focused on healthy tissue sections, clinical translation would face additional challenges, including patient-to-patient variability in autofluorescence, signal interference in complex or pathological tissues, and the practical limitations of the instrumentation. These factors should be considered when assessing potential intraoperative or diagnostic applications.

## 5. Conclusions

This study demonstrates that both MIR- and FTIR-based imaging can reliably cluster chemically and structurally distinct tissue regions, with the spatial resolution being the key determinant of the anatomical accuracy and chemical specificity. Coarse MIR scans alone provided limited structural detail, but integration with high-resolution autofluorescence enabled multimodal clustering that combined chemical contrast with improved spatial accuracy.

The multimodal approach achieves clustering comparable to high-resolution FTIR scans in a fraction of the acquisition time, effectively resolving substructures in the 5–20 µm range and distinguishing lipid-rich white matter from protein-rich gray matter. Compared to H&E staining, it provides rapid, label-free chemical maps over entire tissue sections, complementing conventional histology. Overall, combining high-resolution MIR with autofluorescence offers a time-efficient strategy for tissue characterization, providing a solution that simultaneously delivers high speeds, resolutions, and chemical specificity. Future work should explore additional MIR bands in the fingerprint region to further enhance multimodal analysis.

## Data Availability

The data presented in this study are available on request from the corresponding author.

## Abbreviations

The following abbreviations are used in this manuscript:

MIR	Mid-infrared
IR	Infrared
CT	Computed tomography
H&E	Hematoxylin and eosin
DAPI	Diamidino-phenyl-indole
DNA	Deoxyribonucleic acid
ROI	Region of interest
FTIR	Fourier transform infrared spectroscopy
MALDI	Matrix-assisted laser desorption/ionization
VIS	Visual
NIR	Near-infrared
FAD	Flavins
DFB	Distributed feedback
MS	Megasamples
LED	Light-emitting diode
